# Frequency and implications of malnutrition in systemic sclerosis

**DOI:** 10.1093/rheumatology/keae209

**Published:** 2024-03-28

**Authors:** Jessica L Fairley, Dylan Hansen, Alannah Quinlivan, Susanna Proudman, Joanne Sahhar, Gene-Siew Ngian, Jennifer Walker, Lauren V Host, Kathleen Morrisroe, Wendy Stevens, Laura Ross, Mandana Nikpour

**Affiliations:** Department of Medicine, The University of Melbourne, Melbourne, Victoria, Australia; Department of Rheumatology, St Vincent’s Hospital Melbourne, Melbourne, Victoria, Australia; Department of Rheumatology, St Vincent’s Hospital Melbourne, Melbourne, Victoria, Australia; Department of Medicine, The University of Melbourne, Melbourne, Victoria, Australia; Department of Rheumatology, St Vincent’s Hospital Melbourne, Melbourne, Victoria, Australia; Adelaide Medical School, Faculty of Health and Medical Sciences, University of Adelaide, Adelaide, South Australia, Australia; Department of Rheumatology, Royal Adelaide Hospital, Adelaide, South Australia, Australia; Department of Rheumatology, Monash Health, Melbourne, Victoria, Australia; Department of Medicine, Faculty of Medicine, Nursing and Health Sciences, Monash University, Melbourne, Victoria, Australia; Department of Rheumatology, Monash Health, Melbourne, Victoria, Australia; Department of Medicine, Faculty of Medicine, Nursing and Health Sciences, Monash University, Melbourne, Victoria, Australia; Department of Rheumatology, Royal Adelaide Hospital, Adelaide, South Australia, Australia; Department of Rheumatology, Fiona Stanley Hospital, Perth, Western Australia, Australia; Department of Medicine, The University of Melbourne, Melbourne, Victoria, Australia; Department of Rheumatology, St Vincent’s Hospital Melbourne, Melbourne, Victoria, Australia; Department of Rheumatology, St Vincent’s Hospital Melbourne, Melbourne, Victoria, Australia; Department of Medicine, The University of Melbourne, Melbourne, Victoria, Australia; Department of Rheumatology, St Vincent’s Hospital Melbourne, Melbourne, Victoria, Australia; Department of Medicine, The University of Melbourne, Melbourne, Victoria, Australia; Department of Rheumatology, St Vincent’s Hospital Melbourne, Melbourne, Victoria, Australia; School of Public Health, The University of Sydney, Sydney, New South Wales, Australia; Department of Rheumatology, Royal Prince Alfred Hospital, Sydney, New South Wales, Australia

**Keywords:** malnutrition, systemic sclerosis, body mass index, survival, quality of life

## Abstract

**Objectives:**

To quantify the frequency and impact of malnutrition in systemic sclerosis (SSc), as diagnosed by the Global Leadership Initiative on Malnutrition (GLIM) criteria, based on weight loss, BMI and muscle atrophy.

**Methods:**

Australian Scleroderma Cohort Study participants meeting ACR/EULAR criteria for SSc with ≥1 concurrent weight and height measurement were included. The chi-squared test, two-sample *t*-test or Wilcoxon’s rank-sum test was used for between-group comparison as appropriate. Multivariable logistic regression models were used to establish the determinants of malnutrition diagnosis. Kaplan–Meier and Cox proportional hazard models were used for survival analyses, based on malnutrition diagnosis, and individual GLIM criteria (percentage weight loss, BMI thresholds and presence of muscle atrophy).

**Results:**

In this study of 1903 participants, 43% were diagnosed with malnutrition according to GLIM criteria, of whom 33% had severe malnutrition. Participants diagnosed with malnutrition were older, and more likely to have diffuse cutaneous SSc (dcSSc), higher SSc severity scores and RNA polymerase-3 positivity. Gastrointestinal (GI) involvement, multimorbidity, cardiopulmonary disease, raised inflammatory markers, hypoalbuminaemia and anaemia were more common in malnourished participants (*P* < 0.01). Multimorbidity (odds ratio [OR] 1.6; 95% CI: 1.2, 2.0; *P* < 0.01), pulmonary arterial hypertension (OR 2.1; 95% CI: 1.4, 2.0; *P* < 0.01) and upper GI symptoms (OR 1.6; 95% CI: 1.3, 2.0; *P* < 0.01) were all associated with malnutrition. Health-related quality-of-life (HRQoL) and physical function were poorer in malnourished participants. Survival was worse in those with malnutrition after adjusting for age, sex and dcSSc (hazard ratio 1.4; 95% CI: 1.1, 1.7; *P* < 0.01).

**Conclusion:**

Malnutrition is common in SSc and confers poorer survival, HRQoL and physical function.

Rheumatology key messages

- Forty-three per cent of participants with SSc were diagnosed with malnutrition according to the GLIM criteria.
- Malnutrition was more common with multimorbidity, systemic inflammation, gastrointestinal and cardiopulmonary involvement.
- Poorer health-related quality of life, physical function and survival were observed in malnourished participants.

## Introduction

Malnutrition has no universally accepted definition [1, 2]. It is generally considered to be a deficiency or imbalance of multiple nutrients resulting in adverse changes in body composition, particularly a low body weight for height and/or sarcopenia (the age-related loss of muscle mass) [1, 2]. Malnutrition has been associated with multiple, varied adverse outcomes across different diseases [2–6], including longer hospitalization [5], infections [7] and mortality [5, 8]. Chronic disease is increasingly recognized as an important determinant of nutritional status [2], due to impaired food intake/absorption, and disease-associated chronic inflammation [2]. Risk factors for malnutrition in systemic sclerosis (SSc) include gastrointestinal involvement, diffuse skin disease and increased SSc severity [9]. Limited data suggest that malnutrition may contribute to fatigue, frailty and reduced physical function in SSc [10], and confer worse survival [11]. Identifying those with malnutrition in SSc is thus important in stratifying risk of adverse outcomes, and identifying those patients that may benefit from addressing reversible causes, or from targeted nutrition interventions.

However, reflecting the lack of a universally accepted malnutrition definition in the general population, uncertainty exists about the optimal assessment of nutrition in SSc [12]. The reported frequency of malnutrition varies widely depending on the definition applied. Longitudinal Canadian data suggests that up to 18% of SSc patients are at high risk of malnutrition [9] using the Malnutrition Universal Screening Tool [13] (MUST; Table 1), a screening tool for malnutrition [13] that provides a rapid method for assessing an individual’s malnutrition risk based on unintentional weight loss, BMI and oral intake. However, this tool was designed for use in the inpatient setting [9] so not all criteria can be readily applied to outpatients. Alternately, the Global Leadership Initiative on Malnutrition (GLIM) criteria for malnutrition are designed for both inpatient and outpatient settings [2], with 20–60% [12, 14] of participants diagnosed with malnutrition in cross-sectional SSc cohorts. The GLIM criteria (Table 1) involve both ‘phenotypic’ criteria and ‘aetiological’ criteria, with a positive score across each category required to diagnose malnutrition. The phenotypic criteria include percentage weight loss, reduced BMI, and reduced muscle mass. Aetiological criteria include (i) reduced food intake or assimilation/absorption due to chronic gastrointestinal pathology, or (ii) inflammation, due to either acute illness or a chronic disease associated with mild to moderate chronic/recurrent inflammation [2].

**Table 1.** Summary of the MUST [13] and GLIM [2] criteria, and application in the ASCS

Tool	Parameter	Application to ASCS
MUST: used for malnutrition risk screening		
BMI Score	>20 = 0 points; 18.5–20 = 1 point; <18.5 = 2 points	BMI measured at each visit
Weight loss score	Unplanned weight loss over 3–6 months; <5% = 0 points; 5–10% = 1 point; >10% = 2 points	% weight loss calculated between visits (over 1–2 years)
Acute disease effect score	Patient acutely ill and has had or is likely to have no nutrition intake for >5 days = 2 points	Data unavailable; excluded
Total Score	0 points = low risk; 1 point = medium risk; ≥2 points = high risk of malnutrition	
GLIM criteria for malnutrition diagnosis		
Phenotypic criteria	Weight loss: >5% over 6 months, or >10% over >6 months	Weight loss of >10% over >6 months applied as weight loss calculated between annual study visits
	Low BMI: <20 kg/m^2^ if <70 years, or <22 kg/m^2^ if ≥70 years; or in Asian participants <18.5 kg/m^2^ if <70 years, and <20 kg/m^2^ if ≥70 years	BMI measured at each visit
	Reduced muscle mass: ideally measured by validated body composition techniques, but physical examination can be used if not available	Proximal muscle atrophy identified on examination by treating clinician at each study visit
Aetiological criteria	Reduced food intake or assimilation: ≤50% of energy requirements over >1 week, any reduction over >2 weeks, or any chronic GI condition that adversely impacts food intake or assimilation	All participants meeting ACR/EULAR criteria for SSC assumed to have an aetiologic criteria for malnutrition present
	Inflammation: acute disease/injury or chronic disease-related	
Total score	Malnutrition diagnosed if ≥1 phenotypic and ≥1 aetiological criterion present	
GLIM malnutrition severity	Stage 1 (moderate) malnutrition (1+ of): BMI 18.5–20 kg/m^2^ if <70 years, or 20–22 kg/m^2^ if ≥70 years Weight loss 10–20% over periods longer than 6 months Mild to moderately reduced muscle mass according to validated methods.	BMI measured at each visit
		Weight loss calculated between study visits
		Not applied
	Stage 2 (moderate) malnutrition (1+ of): BMI <18.5 kg/m^2^ if <70 years, or <20 kg/m^2^ if ≥70 years Weight loss >20% over periods longer than 6 months Severely reduced muscle mass according to validated methods	BMI measured at each visit
		Weight loss calculated between study visits
		Not applied

ASCS: Australian Scleroderma Cohort Study; GLIM: Global Leadership Initiative on Malnutrition; MUST: Malnutrition Universal Screening Tool.

Using modified GLIM and MUST tools for diagnosis and risk stratification, respectively, we sought to explore the burden of malnutrition in Australian Scleroderma Cohort Study (ASCS) participants. We aimed to explore the clinical correlates and associations of malnutrition in ASCS participants, and the implications of malnutrition, including effects on survival, physical function and health-related quality of life (HRQoL).

## Methods

Participants were recruited from the multicentre, prospective ASCS. Participants meeting American College of Rheumatology/European Alliance of Associations for Rheumatology (ACR/EULAR) criteria for SSc [15] recruited between 2007 and June 2023 were included. Participants without a concurrent weight and height measurement to facilitate BMI calculation (weight [kilograms] divided by height [metres^2^]) were excluded. The ASCS has been approved by St Vincent’s Hospital Melbourne human research ethics committee (HREC; approval number HREC-A 020/07), and additionally by HRECs at all participating sites, and written informed consent was obtained from all participants.

Demographic and disease data were collected annually. Disease manifestations and autoantibody results were considered present if they had ever been reported since SSc diagnosis. Disease onset was defined as the date of onset of the first non-Raynaud’s phenomenon SSc manifestation. The LeRoy criteria were used to determine disease subtype: diffuse cutaneous (dcSSc) or limited cutaneous (lcSSc) SSc [16]. Where baseline investigation results are presented, this refers to the first-recorded measure within 5 years of recruitment. Presence of symptoms of reflux, vomiting, constipation, diarrhoea, abdominal bloating or faecal incontinence was recorded annually. Oesophageal strictures and dysmotility were considered present based on findings from endoscopy, manometry or barium swallow studies. Small intestinal bacterial overgrowth was diagnosed in the presence of concurrent bloating and diarrhoea/incontinence, or use of cyclic antibiotics for treatment. Lower gastrointestinal dysmotility was diagnosed on nuclear medicine studies. Ischaemic heart disease (IHD) was defined as patient-reported angina or myocardial infarction, coronary artery bypass grafting or stenting. Pulmonary arterial hypertension (PAH) was defined by right heart catheterization findings according to the revised PAH classification criteria [17] or previous classification criteria [18]. Interstitial lung disease (ILD) was diagnosed using high-resolution CT (HRCT) of the chest performed at physician discretion in response to clinical assessment or abnormal pulmonary function test results, with severity defined by the extent of radiological involvement and percent-predicted forced vital capacity (FVC) (limited: <20% HRCT involvement or 20–30% with FVC ≥ 70%; or extensive: >30% HRCT involvement or 20–30% with FVC < 70%) [19]. Current medications, the presence of co-morbid diabetes, dyslipidaemia and hypertension, and smoking were recorded from patient-reported history and medical record review at each study visit. Scleroderma renal crisis (SRC) was diagnosed in the presence of two of three criteria: new-onset hypertension with no alternative cause, unexplained rise in serum creatinine or microangiopathic haemolytic anaemia. Creatine kinase (CK) elevation was defined as >140 IU/l, CRP elevation as >5 IU/l, hypoalbuminaemia as <35 g/l, and anaemia as haemoglobin levels <120 g/l. Proximal weakness was defined as reduced power (<5/5) on clinical examination. Oral aperture was measured annually on examination.

At each study visit, the World Health Organization (WHO) Functional Class was recorded by the study physician. Patient-reported outcome measures (PROMs) were collected annually, including Health Assessment Questionnaire Disability Index (HAQ-DI), Functional Assessment of Chronic Illness Therapy-Fatigue (FACIT), Short Form-36 (SF-36) and University of California Los Angeles (UCLA) Scleroderma Clinical Trials Consortium (SCTC) Gastrointestinal Tract (GIT) scores. The Medsger Severity Score (MSS) was calculated to assess the overall SSc burden at each study visit [20]. To measure multimorbidity at each study visit, we calculated a modified Charlson Comorbidity Index (CCI) score [21]. A list of included items is provided in Supplementary Table S1, available at *Rheumatology* online; data for some variables (including hemiplegia, HIV/AIDS and dementia) were excluded as these data are not collected as part of the ASCS protocol. A CCI score ≥4 was defined as a significant comorbidity burden [22], with the highest available score being 19.

### Malnutrition assessments

The criteria and application of both the MUST and GLIM criteria are summarized in Table 1. In brief, MUST scores were used to estimate malnutrition risk, calculated as the sum of a score allocated for percentage weight loss over one to two visits (0–2 points allocated depending on percentage of weight loss) and an additional score allocated for BMI category (0–2 points depending on BMI). We do not routinely collect data about oral intake in the 5 days leading up to the study visit, so this MUST criterion could not be applied. The MUST score was only calculated when both serial weight measurements and BMI were available to avoid underestimating malnutrition frequency. Total scores ≥2 represented high malnutrition risk, 1 moderate risk, and 0 low risk. Three GLIM phenotypic criteria were applied: weight loss, low BMI and reduced muscle mass. ASCS participants are reviewed annually, so weight loss between visits was used to calculate percentage weight change. Low BMI was measured at each visit and defined as <20 kg/m^2^ in those <70 years of age, and <22 kg/m^2^ in those ≥70 years of age. At each study visit the clinician assessed for the presence of proximal muscle atrophy on clinical examination, which was defined as present or absent; grading of severity of muscle wasting was not available. Formal measurements of body composition (anthropometric measures) were not available. Given that SSc is a severe multisystem disease associated with a high burden of gastrointestinal symptoms, chronic recurrent inflammation and severe cardiopulmonary conditions that predispose to malnutrition, we considered that all participants meeting ACR/EULAR criteria for SSc fulfilled aetiological criteria for malnutrition. Malnutrition was considered present if ≥1 phenotypic criterion was present at any time. Participants were then grouped into those who had ever been diagnosed with malnutrition according to GLIM criteria, and those who were not malnourished. Malnutrition severity was estimated by applying GLIM Criteria for malnutrition severity, based on BMI and percentage weight loss alone as the severity of muscle atrophy is not recorded in the ASCS (Table 1).

### Statistical analysis

Characteristics of study participants are presented as mean (s.d.) for normally distributed continuous variables, median (interquartile range) for non-normally distributed continuous variables, and as a number (percentage) for discrete variables. Comparisons between groups were performed using Student’s two-sample *t*-test for normally distributed continuous variables, Wilcoxon’s rank sum test for non-normally distributed continuous variables and the chi-squared test for discrete variables. Logistic regression analysis was performed to explore clinical associations in those with and without GLIM malnutrition, with covariates for multivariable models selected based on clinical and statistical significance. Collinear covariates were excluded. Given the potential inverse association between oral aperture and malnutrition, for analysis we centred oral aperture measurements around the mean oral aperture (cm), by subtracting the measured oral aperture from the cohort mean to describe the odds of malnutrition for each cm reduction in oral aperture. Survival analysis was performed using the end point of all-cause mortality. Kaplan–Meier survival curves and Wilcoxon’s test were used to estimate survival from SSc onset. Multivariable Cox proportional hazards regression modelling was used to determine multivariable determinants of mortality. Covariates were chosen for the multivariable analysis if they were either clinically relevant or statistically significant on univariable analysis (*P* *<* 0.05) and did not violate the proportional hazards assumption. Results are reported as hazard ratios (HR) with accompanying 95% CI. Analysis was performed using STATA 17.0 (StataCorp LLC, College Station, TX, USA).

## Results

### Predicting malnutrition in systemic sclerosis

Of 1903 included participants, 811 (42.6%) met GLIM criteria for a diagnosis of malnutrition. Using the GLIM criteria to assess malnutrition severity (Table 1), 373 participants (46.0%) had stage 1 (moderate) malnutrition while 268 participants (33.1%) were severely (stage 2) malnourished. Malnutrition was non-severe in 170 participants (21.0%).

**Figure 1.**
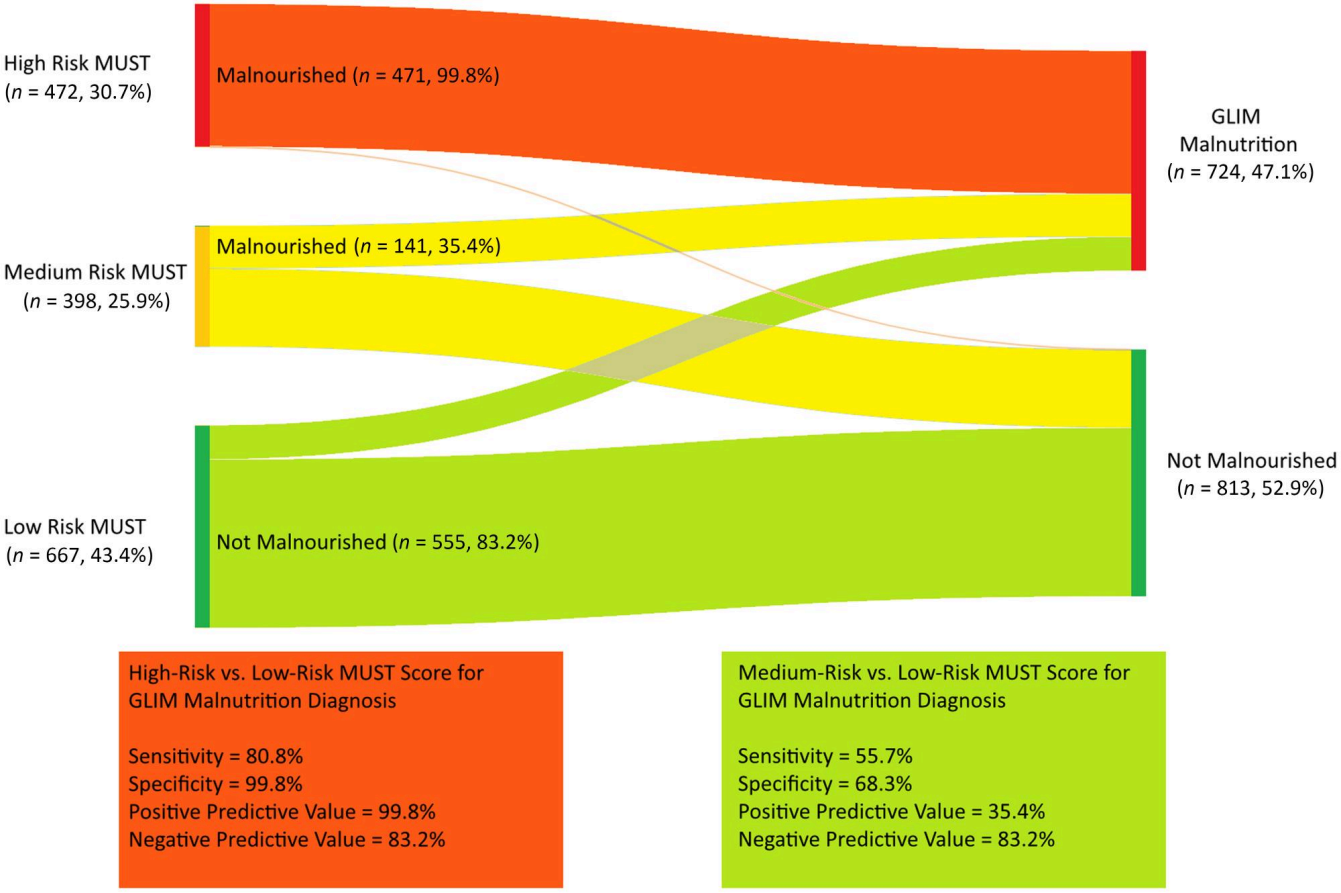
MUST malnutrition Risk and GLIM malnutrition diagnosis in included participants (*n* = 1537). GLIM: Global Leadership Initiative on Malnutrition; MUST: Malnutrition Universal Screening Tool

Malnutrition risk using the modified MUST could be estimated in 1537 participants (80.8%) who had serial weight measurements recorded a maximum of two visits apart. Of these participants, 472 were considered to be at high risk of malnutrition ever (30.7%), 398 (25.9%) at moderate risk and 667 (43.4%) at low risk of malnutrition (Fig. 1). Of those with GLIM-criteria malnutrition and an estimable modified MUST score, 471 (65.1%) had high-risk MUST scores, 141 (19.5%) had medium-risk modified MUST scores and 112 (15.5%) had low-risk modified MUST scores (Fig. 1; Supplementary Table S2, available at *Rheumatology* online). Only one participant without malnutrition was identified as high-risk using the modified MUST tool due to a BMI recording of 19.9 kg/m^2^, which did not meet the threshold for malnutrition for Asian participants according to the GLIM criteria (Table 1). Accordingly, the positive predictive value (PPV) of a high-risk compared with a low-risk modified MUST score for malnutrition diagnosis was 99.8%, with a negative predictive value (NPV) of 83.2%, a sensitivity of 80.8% and specificity of 99.8%. For a medium-risk compared with a low-risk modified MUST score, the PPV was 35.4%, with a NPV of 83.2%, sensitivity of 55.7% and a specificity of 68.3%.

### Characteristics of participants with GLIM malnutrition

Participants identified as having malnutrition were older (*P* = 0.01) with longer follow-up (*P* *<* 0.01) but a similar disease duration at first study visit (*P* *=* 0.86; Table 2). As expected, lower baseline BMI (*P < *0.01) and highest-recorded percentage weight loss (*P* *<* 0.01) were observed in those with malnutrition. While participants who had been obese were less likely to be malnourished, 22.8% were still identified as ever being malnourished (*P* *<* 0.01). Proximal muscle atrophy was present in 50.2% of those with malnutrition. Malnourished participants were more likely to have dcSSc (*P* *<* 0.01) and had higher baseline SSc severity scores (*P* *<* 0.01), with no difference in sex or smoking status. RNA polymerase-3 positivity was more common in those with malnutrition, while ANA centromere positivity was less common (*P* *<* 0.01).

**Table 2.** Description of the included cohort

Variable^a^	Overall cohort (*n* = 1903; 100%)	GLIM-diagnosed malnutrition (*n* = 811; 42.6%)	Never malnourished (*n* = 1092; 57.4%)	*P*-value
Age at SSc onset, median (IQR), years (*n* = 1796)	47.3 (36.4–57.0)	48.2 (36.8–59.0)	46.8 (36.2–55.5)	0.01
Male sex, *n* (%) (*n* = 1903)	272 (14.3)	122 (15.0)	150 (13.7)	0.42
Diffuse cutaneous SSc, *n* (%) (*n* = 1903)	508 (26.7)	249 (30.7)	259 (23.7)	<0.01
Non-Caucasian ethnicity, *n* (%) (*n* = 1807)	158 (8.7)	65 (8.4)	93 (9.0)	0.65
Disease duration at baseline visit, median (IQR), years (*n* = 1796)	7.2 (2.6–15.7)	6.9 (2.4–16.2)	7.5 (2.7–15.3)	0.86
Follow-up, median (IQR), years (*n* = 1903)	4.5 (1.6–8.6)	5.7 (2.7–9.4)	3.5 (1.0–7.6)	<0.01
Died, *n* (%) (*n* = 1901)	385 (20.3)	249 (30.7)	136 (12.5)	<0.01
MRSS (highest)^l^, median (IQR) (*n* = 1868)	8 (5–16)	9 (5–19)	8 (4–14)	<0.01
Smoker^l^, *n* (%) (*n* = 1903)	951 (50.0)	420 (51.8)	531 (48.6)	0.17
MSS Score (baseline), median (IQR) (*n* = 1903)	5 (3–7)	6 (4–8)	5 (3–7)	<0.01
Multimorbidity^a^^,^^l^, *n* (%) (*n* = 1903)	458 (24.1)	253 (31.2)	205 (18.8)	<0.01
BMI (lowest)^l^, median (IQR), kg/m^2^ (*n* = 1903)	24.1 (20.9–27.5)	20.9 (18.8–24.4)	25.8 (23.2–29.0)	<0.01
BMI (baseline^m^^,^^l^), median (IQR), kg/m^2^ (*n* = 1903)	25.6 (22.4–29.3)	23.5 (20.3–27.9)	26.6 (23.8–30.1)	<0.01
Obesity (BMI ≥ 30 kg/m^2^)^l^, *n* (%) (*n* = 1903)	534 (28.1)	185 (22.8)	349 (32.0)	<0.01
Weight loss (highest)^l^, median (IQR), % (*n* = 1540)	5.1 (2.0–9.7)	10 (4.7–14.1)	3.3 (0.7–5.7)	<0.01
Muscle atrophy^l^, *n* (%) (*n* = 1882)	407 (21.4)	407 (50.2)	0 (0.0)	<0.01
Serology, *n* (%)				
ANA positive (*n* = 1861)	1778 (95.5)	761 (95.0)	1017 (95.5)	0.33
ANA centromere (*n* = 1847)	854 (46.2)	339 (42.1)	515 (49.5)	<0.01
ENA				
Scl70 (*n* = 1819)	268 (14.7)	119 (15.2)	149 (14.4)	0.64
RNA polymerase-3 (*n* = 1779)	182 (14.1)	102 (17.3)	80 (11.4)	<0.01
Gastrointestinal symptoms				
UCLA GIT scores (highest reported)^l^, median (IQR) (*n* = 908)	0.5 (0.2–1.0)	0.6 (0.3–1.1)	0.5 (0.2–0.9)	<0.01
Sicca symptoms^l^, *n* (%) (*n* = 1903)	1523 (80.0)	685 (84.5)	838 (76.7)	<0.01
Dysphagia^l^, *n* (%) (*n* = 1592)	994 (62.4)	510 (68.9)	484 (56.8)	<0.01
Reflux^l^, *n* (%) (*n* = 1887)	1608 (85.2)	712 (88.2)	896 (83.0)	<0.01
Vomiting^l^, *n* (%) (*n* = 1889)	493 (26.1)	276 (34.2)	217 (20.0)	<0.01
Faecal incontinence^l^, *n* (%) (*n* = 1892)	632 (33.4)	323 (40.0)	209 (28.5)	<0.01
Diarrhoea^l^, *n* (%) (*n* = 1893)	1022 (54.0)	490 (60.6)	532 (49.1)	<0.01
Constipation^l^, *n* (%) (*n* = 1890)	1004 (53.1)	467 (57.9)	537 (49.6)	<0.01
Gastrointestinal manifestations, *n* (%)				
Oesophageal stricture^b^^,^^l^ (*n* = 1903)	205 (10.8)	120 (14.8)	85 (7.8)	<0.01
SIBO^c^^,^^l^ (*n* = 1903)	779 (40.9)	370 (45.6)	409 (37.5)	<0.01
Oesophageal dysmotility^d^^,^^l^ (*n* = 1903)	228 (12.0)	121 (14.9)	107 (9.8)	<0.01
Bowel dysmotility^e^^,^^l^ (*n* = 1903)	89 (4.7)	54 (6.7)	35 (3.2)	<0.01
Pseudo-obstruction^l^ (*n* = 1752)	69 (3.9)	36 (4.7)	33 (3.4)	0.17
Other SSc features or comorbidities, *n* (%)				
Ischaemic heart disease^f^^,^^l^ (*n* = 1897)	272 (14.3)	154 (19.1)	118 (10.8)	<0.01
LVEF < 50%^l^ (*n* = 1786)	96 (5.4)	59 (7.6)	37 (3.7)	<0.01
PAH^l^ (*n* = 1903)	182 (9.6)	117 (14.4)	65 (6.0)	<0.01
ILD (HRCT)^g^^,^^l^ (*n* = 1903)	537 (28.2)	275 (33.9)	262 (24.0)	<0.01
Raynaud’s phenomenon^l^ (*n* = 1903)	1886 (99.1)	807 (99.5)	1079 (98.8)	0.10
Digital ulcers^l^ (*n* = 1903)	1023 (53.8)	516 (63.6)	507 (46.4)	<0.01
Tendon friction rubs^l^ (*n* = 1903)	172 (9.0)	114 (14.1)	58 (5.3)	<0.01
Synovitis^l^ (*n* = 1903)	786 (41.3)	370 (45.6)	416 (38.1)	<0.01
SSc renal crisis^l^ (*n* = 1903)	68 (3.6)	48 (5.9)	20 (1.8)	<0.01
Proximal weakness^h^^,^^l^ (*n* = 1876)	412 (22.0)	278 (34.7)	134 (12.5)	<0.01
Albumin < 35g/l^l^ (*n* = 1854)	518 (27.9)	305 (38.1)	213 (20.2)	<0.01
Anaemia (haemoglobin < 120g/l)^l^ (*n* = 1860)	727 (39.1)	435 (54.2)	292 (27.6)	<0.01
CRP > 5IU/l^l^ (*n* = 1824)	953 (52.2)	484 (61.2)	469 (45.4)	<0.01
CK elevation (≥140IU/l)^l^ (*n* = 1792)	574 (32.0)	274 (35.2)	300 (29.6)	0.01
Physical function and HRQoL^j^				
WHO class III/IV dyspnoea^l^, *n* (%) (*n* = 1852)	627 (33.9)	369 (46.3)	258 (24.5)	<0.01
HAQ-DI (highest)^l^, median (IQR) (*n* = 1542)	1 (0.4–1.6)	1.3 (0.6–1.9)	0.8 (0.3–1.4)	<0.01
SF-36 PCS (lowest)^l^, median (IQR) (*n* = 1561)	30.9 (24.0–41.3)	27.8 (21.9–36.9)	34.3 (26.3–44.0)	<0.01
SF-36 MCS (lowest)^l^, median (IQR) (*n* = 1561)	40.4 (31.0–50.5)	38.5 (29.6–48.0)	42.5 (32.0–51.5)	<0.01
FACIT-Fatigue (lowest)^l^, median (IQR) (*n* = 896)	22.3 (11–35.5)	21 (10–32)	23.6 (12–38)	<0.01
Treatments, *n* (%)				
Immunosuppression exposure^k^^,^^l^ (*n* = 1903)	1137 (59.7)	534 (65.8)	603 (55.2)	<0.01
Prednisolone^l^ (*n* = 1903)	876 (46.0)	432 (53.3)	444 (40.7)	<0.01
Mycophenolate^l^ (*n* = 1903)	262 (13.8)	141 (17.4)	121 (11.1)	<0.01
Methotrexate^l^ (*n* = 1903)	465 (24.4)	225 (27.7)	240 (22.0)	<0.01
Rituximab^l^ (*n* = 1903)	34 (1.8)	19 (2.3)	15 (1.4)	0.11
Tocilizumab^l^ (*n* = 1903)	14 (0.7)	7 (0.9)	7 (0.6)	0.58
Cyclophosphamide^l^ (*n* = 1903)	158 (8.3)	89 (11.0)	69 (6.3)	<0.01
Calcium channel blocker^l^ (*n* = 1903)	1266 (66.5)	579 (71.4)	687 (62.9)	<0.01
Vitamin D supplementation^l^ (*n* = 1903)	716 (37.6)	351 (43.3)	365 (33.4)	<0.01
Proton pump inhibitor^l^ (*n* = 1903)	1583 (83.2)	700 (86.3)	883 (80.9)	<0.01
TPN^l^ (*n* = 1903)	7 (0.4)	7 (0.9)	0 (0)	<0.01
Cyclic Abx for SIBO^l^ (*n* = 1903)	160 (8.4)	88 (10.9)	72 (6.6)	<0.01

a Number of variables (*n*) available for analysis presented.

b Multimorbidity defined as Charlson Comorbidity Index Scores ≥4.

c Oesophageal stricture defined on endoscopy.

d SIBO defined as concurrent abdominal bloating and diarrhoea/incontinence, or use of cyclic antibiotics for treatment.

e Oesophageal dysmotility defined on manometry or nuclear medicine studies.

f Bowel dysmotility defined on nuclear medicine studies.

g IHD defined as patient-reported ischaemic chest pain or abnormal coronary angiography.

h ILD diagnosed on HRCT Chest.

i Proximal weakness defined as power <5/5 on physical examination.

j Highest or lowest patient-reported outcome measure value reported depending on whether higher or lower value represents worse outcome on a given tool.

k Immunosuppression exposure defined as use of corticosteroid, synthetic or biologic immunosuppression.

l Denotes ever recorded from SSc onset.

m Where data from baseline visit missing, first-recorded value from first 5 visits used.

ANA: antinuclear antibody; CK: creatine kinase; ENA: extractable nuclear antigen; FACIT: functional assessment of chronic illness therapy; GIT: gastrointestinal tract; GLIM: Global Leadership Initiative on Malnutrition; HAQ-DI: health assessment questionnaire disability index; HRCT: high-resolution CT; HRQoL: health-related quality of life; ILD: interstitial lung disease; IQR: interquartile range; LVEF: left ventricular ejection fraction; MCS: mental composite summary; MRSS: modified Rodnan Skin Score; PAH: pulmonary arterial hypertension; PCS: physical composite summary; SF-36: Short Form Survey-36; SIBO: small intestinal bacterial overgrowth; SSc: systemic sclerosis; TPN: total parenteral nutrition; UCLA: University of California Los Angeles; WHO: World Health Organization.

Those with malnutrition had more severe GI involvement as measured by the UCLA SCTC GIT 2.0 Score (*P* *<* 0.01). There was an increased prevalence of GI symptoms such as dysphagia, reflux and vomiting (*P* *<* 0.01) in patients with malnutrition and an increased frequency of GIT damage in this group with a higher prevalence of confirmed oesophageal or lower GI dysmotility as well as oesophageal strictures (*P* *<* 0.01). Pseudo-obstruction was numerically more common in this group, although this did not meet statistical significance (*P* *=* 0.17).

Multimorbidity was more common in those with malnutrition (*P* *<* 0.01), including severe cardiopulmonary disease such as IHD, reduced left ventricular function, PAH and ILD (*P* *<* 0.01; Table 2). Digital ulceration, tendon friction rubs, synovitis and SRC were all more common in this group (*P* *<* 0.01). Proximal muscle weakness (*P* *<* 0.01) and CK elevation (*P* *=* 0.01) were more common in those with malnutrition. Features indicating a chronic inflammatory state such as raised CRP, hypoalbuminaemia and anaemia were more common in this group (all *P* *<* 0.01). Those with malnutrition were more likely to have received immunosuppression including prednisolone, methotrexate, mycophenolate and cyclophosphamide (*P* *<* 0.01), with small numbers only receiving rituximab or tocilizumab. Those with malnutrition were also more likely to have received calcium channel antagonists and vitamin D supplementation (*P* *<* 0.01).

### Malnutrition and HRQoL

General HRQoL was poorer in this group as measured by SF-36 Physical Component Summary scores (*P* *<* 0.01) and FACIT-Fatigue Scores (*P* *<* 0.01), as was mental wellbeing measured by SF-36 Mental Component Summary scores (*P* *<* 0.01). Those with malnutrition reported increased functional disability as measured by the HAQ-DI (*P* *<* 0.01), and higher WHO Functional Class (*P* *<* 0.01).

### Survival

**Figure 2.**
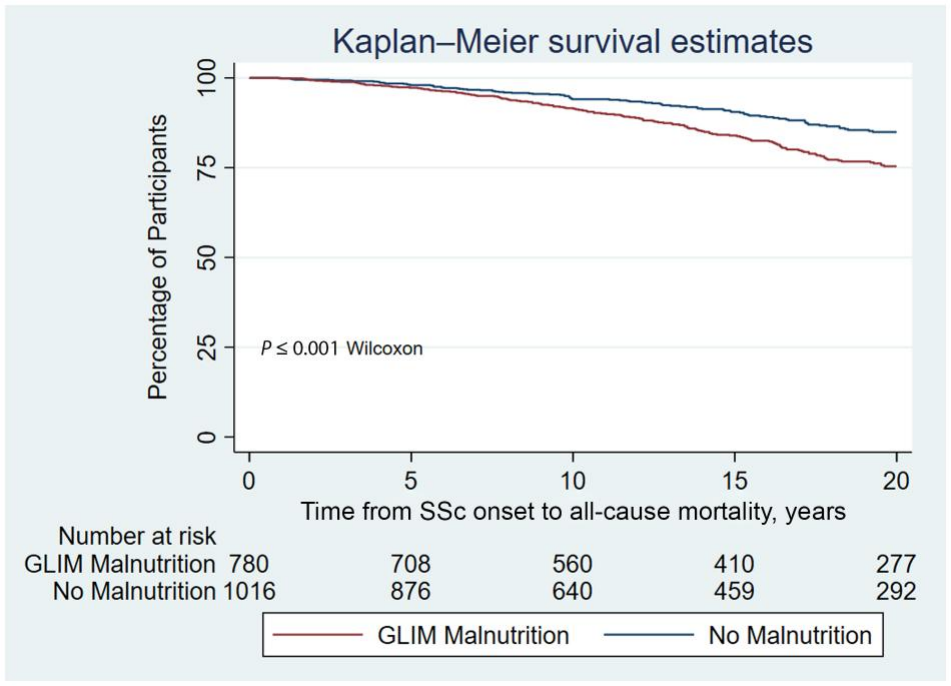
Kaplan–Meier survival analysis comparing those with and without GLIM-diagnosed malnutrition. GLIM: Global Leadership Initiative on Malnutrition; MUST: Malnutrition Universal Screening Tool

Those with malnutrition had worse survival than those without (Fig. 2). GLIM malnutrition was associated with worse survival (HR 1.4; 95% CI: 1.1, 1.7; *P* *<* 0.01) adjusting for age at SSc onset (HR 1.1; 95% CI: 1.1, 1.1; *P* *<* 0.01), male sex (HR 2.1; 95% CI: 1.6, 2.7; *P* *<* 0.01) and dcSSc (HR 2.2; 95% CI: 1.8, 2.8; *P* *<* 0.01) (Table 3; univariate analysis in Supplementary Table S3, available at *Rheumatology* online). Multivariable analysis of the individual components of the GLIM criteria, adjusting for age at SSc onset, male sex and dcSSc, showed that all individual GLIM criteria were also associated with reduced survival (BMI < 20 kg/m^2^ [2]; HR 1.4; 95% CI: 1.1, 1.7; *P* *<* 0.01), muscle atrophy (HR 1.5; 95% CI: 1.2, 1.8; *P* *<* 0.01) and weight loss >10% (HR 1.6; 95% CI: 1.2, 2.2; *P* *<* 0.01). Annual weight loss of between 5 and 10% had no effect on overall survival (HR 1.0; 95% CI: 0.8, 1.3; *P* *=* 0.97). Findings of a sensitivity analysis exploring the prognostic impact of malnutrition in those with incident SSc (≤5 years from SSc onset to ASCS recruitment) were similar, except for an attenuation of the prognostic impact of muscle atrophy (*P* *=* 0.06) (Supplementary Table S4, available at *Rheumatology* online).

**Table 3.** Multivariable Cox proportional hazard model for survival in those with malnutrition

Variable	Hazard ratio	95% CI	*P*-value
Model using GLIM malnutrition diagnosis (*n* = 1796)			
GLIM Malnutrition^a^	1.4	1.1, 1.7	<0.01
Age at SSc onset (years)	1.1	1.1, 1.1	<0.01
Male sex	2.1	1.6, 2.7	<0.01
Diffuse SSc	2.2	1.8, 2.8	<0.01
Model using BMI (*n* = 1796)			
BMI <20 kg/m^2^^a^	1.4	1.1, 1.7	<0.01
Age at SSc onset (years)	1.1	1.1, 1.1	<0.01
Male sex	2.1	1.7, 2.7	<0.01
Diffuse SSc	2.3	1.8, 2.8	<0.01
Model using weight loss (highest recorded, %) (*n* = 1467)			
Weight loss <5%^a^	—	—	—
Weight loss 5–10%^a^	1.0	0.8, 1.3	0.97
Weight loss >10%^a^	1.6	1.2, 2.2	<0.01
Age at SSc onset (years)	1.1	1.1, 1.1	<0.01
Male sex	2.1	1.6, 2.9	<0.01
Diffuse SSc	2.1	1.7, 2.8	<0.01
Model using muscle atrophy (*n* = 1777)			
Muscle atrophy^a^	1.5	1.2, 1.8	<0.01
Age at SSc onset (years)	1.1	1.1, 1.1	<0.01
Male sex	2.0	1.6, 2.6	<0.01
Diffuse SSc	2.2	1.7, 2.7	<0.01

a Denotes ever from SSc onset. GLIM: Global Leadership Initiative on Malnutrition.

### Associations of malnutrition

In a multivariable logistic regression model exploring the association of GLIM malnutrition (Table 4; univariate analyses in Supplementary Table S5, available at *Rheumatology* online), multimorbidity (OR 1.6; 95% CI: 1.2, 2.0; *P < *0.01), and vomiting or dysphagia (OR 1.6; 95% CI: 1.3, 2.0; *P* *<* 0.01) were associated with malnutrition. Each centimetre reduction in oral aperture below the cohort mean was also associated with an increase in malnutrition (OR 1.2; 95% CI: 1.0, 1.3; *P* *=* 0.01). Diarrhoea or faecal incontinence (OR 1.2; 95% CI: 1.0, 1.5; *P* *=* 0.08) and dcSSc (OR 1.2; 95% CI: 1.0, 1.5; *P* *=* 0.08) were also associated with malnutrition although not reaching statistical significance. Digital ulceration (OR 1.6; 95% CI: 1.3, 1.9; *P* *<* 0.01), raised CRP (OR 1.4; 95% CI: 1.1, 1.7; *P* *=* 0.01) and PAH (OR 2.0; 95% CI: 1.4, 3.0; *P* *<* 0.01) were associated with increased risk of malnutrition. Extensive ILD was also associated with malnutrition although this did not reach statistical significance (OR 1.4; 95% CI: 1.0, 2.0; *P* *=* 0.07), while limited ILD was not (*P* *=* 0.74).

**Table 4.** Multivariable logistic regression model for determinants of GLIM-criteria malnutrition (*n* = 1673)

Variable	OR	**95% CI**	*P*-value
Diffuse SSc	1.2	1.0–1.5	0.11
Multimorbidity^a^^,^^b^	1.6	1.2–2.0	<0.01
Vomiting/dysphagia^a^^,^^c^	1.6	1.3–2.0	<0.01
Diarrhoea/incontinence^a^^,^^d^	1.2	1.0–1.5	0.08
Oral Aperture (cm, lowest recorded)^a^^,^^e^	1.2	1.0–1.3	0.01
Digital ulceration^a^	1.6	1.3–1.9	<0.01
CRP > 5 IU/l^a^	1.4	1.1–1.7	<0.01
PAH^a^	2.0	1.4–3.0	<0.01
ILD^a^			
Limited ILD^f^	1.0	0.8–1.4	0.74
Extensive ILD^f^	1.4	1.0–2.0	0.07

a Denotes ever from SSc onset.

b Multimorbidity defined as Charlson Comorbidity Index Scores ≥4.

c Symptoms of patient-reported vomiting or dysphagia combined due to strong association with malnutrition, and strong correlation between variables.

d Symptoms of patient-reported diarrhoea or faecal incontinence combined due to strong association with malnutrition, and strong correlation between variables.

e Lowest-recorded value of oral aperture centred around mean value to facilitate meaningful odds ratio calculation (mean oral aperture minus measured oral aperture value), to describe the increase in malnutrition risk with each cm below mean oral aperture.

f Limited-stage ILD defined as <20% HRCT involvement, or 20–30% involvement with percent-predicted forced vital capacity ≥ 70%, while extensive-stage ILD defined as ≥30% HRCT extent, or 20–30% HRCT involvement with percent-predicted forced vital capacity < 70%. GLIM: Global Leadership Initiative on Malnutrition; ILD: interstitial lung disease; OR: odds ratio; PAH: pulmonary arterial hypertension.

## Discussion

Over 40% of a large SSc cohort met the GLIM diagnostic criteria for malnutrition, with 20% identified as high risk of malnutrition using the modified MUST screening tool. Malnutrition was associated with poorer survival and reduced HRQoL. To our knowledge, this is the largest SSc cohort to which the GLIM criteria have been applied. In smaller, cross-sectional SSc cohorts undergoing anthropometric testing, between 20% [14] and 60% [12] of participants were diagnosed with GLIM-criteria malnutrition. Of note, these criteria are highly influenced by weight loss: in the cross-sectional study reporting a 20% malnutrition prevalence, median percentage weight loss was only 0.5% [14], compared with 5.1% in our longitudinal cohort with 5 years’ follow-up. Furthermore, those with malnutrition in our cohort had a longer follow-up, suggesting that risk of malnutrition in SSc may be cumulative. We have demonstrated a similar proportion (around 20%) of high-risk MUST scores compared with other longitudinal cohorts [9, 23]. Regardless of the definition applied, these data suggest malnutrition is highly prevalent in SSc, and may be underestimated in cross-sectional data.

While the GLIM criteria are used to diagnose malnutrition, the MUST tool assesses risk of malnutrition [2, 13]. Comparing the modified MUST and the GLIM criteria, we showed that most participants diagnosed with GLIM malnutrition had high- or medium-risk MUST scores. However, one in six participants with low-risk modified MUST scores met GLIM criteria for malnutrition, and only one-third of those with medium-risk modified MUST scores met GLIM criteria for malnutrition. Given that the MUST score is designed for use in inpatient cohorts and not all criteria are readily applicable in outpatients, it follows that these criteria may be inaccurate or underestimate the true prevalence of malnutrition in SSc. Optimal methods of screening or prediction of those SSc patients at increased risk of malnutrition are yet to be validated. We have shown that high-risk modified MUST scores had a PPV of 99.8% and NPV of 80.8% for predicting malnutrition compared with low-risk modified MUST scores, while medium-risk modified MUST scores had a PPV of 35.4% and NPV of 83.2%. This suggests that while high- or low-risk modified MUST scores may be useful in identifying malnutrition, medium-risk scores appear less useful. Anthropometric measures to assess nutrition may help to stratify this medium-risk category; however, they are not routinely collected in clinical practice.

Malnourished participants experienced worse survival, including after adjusting for age, sex and dcSSc, confirming findings in smaller and cross-sectional cohorts suggesting poorer prognosis in malnourished SSc patients [11, 24]. In addition to malnutrition diagnosis, >10% weight loss, a BMI lower than 20 kg/m^2^ and muscle atrophy were all identified to be individually important negative prognostic factors. This suggests that each of these components of malnutrition diagnosis confer individual prognostic importance, with a BMI threshold of 20 kg/m^2^ and weight loss threshold of 10% being of particular importance. Other data in smaller cohorts have not suggested an association of BMI or weight loss with survival in SSc [24], despite a negative prognostic impact of malnutrition diagnosis. However, our longitudinal data indicate that these simple, easily recordable biomarkers may have a role in identifying those at increased risk of adverse outcomes. Relying on weight loss and BMI thresholds alone to screen for malnutrition in SSc may result in delay to diagnosis and intervention, especially given our finding that these are individually associated with adverse prognosis.

Importantly, we also identified universally reduced HRQoL in malnourished participants. This group had a higher burden of gastrointestinal symptoms, fatigue and dyspnoea, experiencing worse functional disability and physical and mental wellbeing. This confirms preliminary cross-sectional data in people with SSc that suggested lower HRQoL in those with SSc and high-risk MUST scores [10].

We report multiple important associations of malnutrition in SSc that support the likely multifactorial nature of nutritional disturbance in these patients. GI involvement (particularly of the upper GIT) has been consistently associated with malnutrition [9, 25], weight loss [26] and micronutrient deficiencies [27] in SSc, likely the result of reduced food absorption/assimilation and possibly restricted food intake or change in composition of food. However, this study identifies important extra-gastrointestinal associations of malnutrition that raise interesting additional potential mechanisms of nutritional disturbance in SSc. We report the novel association of multimorbidity and malnutrition diagnosis in SSc. While malnutrition has been associated with increased comorbidity burden in the general population [28], this association has not previously been demonstrated in SSc. Multiple cardiopulmonary diseases were more frequent in those with malnutrition, including PAH and extensive ILD. Lung involvement in SSc has been associated with malnutrition [25], and PAH with both micronutrient deficiency and malnutrition risk in SSc [27]. This supports the idea that advanced respiratory disease has secondary effects on multiple organ systems, particularly malnutrition and metabolic disturbance. Biochemical inflammation is purported to alter body composition as well as increasing anorexia and thus reducing food intake [2], with raised inflammatory markers associated with malnutrition in our cohort. Moreover, we identified an increase in use of immunosuppression including corticosteroids, methotrexate, mycophenolate and cyclophosphamide in malnourished participants. Both corticosteroids and specific immunosuppressive agents (e.g. mycophenolate mofetil) may exacerbate GI symptoms in SSc and thus reduce appetite or enteric absorption, or act be an indicator of more severe disease/chronic inflammation. dcSSc was associated with malnutrition although significance was attenuated in multivariable modelling; other data have identified Scl70 positivity as a predictor of malnutrition in SSc [29]. Finally, digital ulceration was associated with malnutrition; whether this is because malnutrition adversely impacts wound healing, that digital ulceration renders food preparation more difficult, a decrease in appetite due to pain, or due to other factors is uncertain. These data highlight the multiple potential mechanisms of nutritional disturbance in SSc that may contribute to reduced food intake/appetite, reduced absorption and altered body composition resulting in malnutrition.

This study has limitations. We did not have anthropometric measures available to confirm the presence of muscle atrophy. Muscle atrophy can be a non-specific finding and may occur for multiple reasons in SSc, including myopathy and cardiopulmonary disease, which may confound our findings. However, we have used international consensus criteria for malnutrition diagnosis and the authors of these guidelines acknowledge that anthropometric measures supporting a diagnosis of malnutrition are not always available and examination findings can be used in their absence [2]. Some participants did not have weight measurements over consecutive visits, which limited the number of participants with calculable MUST scores. Furthermore, a minority of patients (*n* = 21) did not have proximal muscle atrophy recorded. Together, this means we may have underestimated the frequency of malnutrition in our cohort, and thus underestimated the impact of malnutrition in our cohort. Our cohort is predominantly a prevalent cohort of SSc, with those with rapid onset and deterioration less likely to survive to recruitment. Accordingly, there is significant survivor bias in this study population. Finally, our cohort is predominantly Caucasian limiting generalizability to other racial groups. This is particularly important when considering malnutrition as an outcome given that BMI thresholds may not be valid in non-Caucasian cohorts. Some variables had incomplete outcome data.

## Conclusions

Over 40% of our large SSc cohort met diagnostic criteria for malnutrition, with one-third of malnourished participants having severe malnutrition. Gastrointestinal symptoms, PAH, biochemical inflammation and multimorbidity were associated with malnutrition. Malnutrition was associated with increased mortality, universally poorer HRQoL and a higher symptom burden. These data highlight both the frequency and importance of malnutrition in SSc, warranting consideration of early referral to dietetic and clinical nutrition services. Further data are required to explore whether malnutrition is reversible and associated with improved outcomes if successfully treated.

## Supplementary Material

keae209_Supplementary_Data

## Data Availability

The data underlying this article are available in the article and its online supplementary material.
