# Supplementary material for: Frequency and implications of malnutrition in systemic sclerosis
Source: Rheumatology (Oxford). 2024 Mar 28;64(3):1251–60. doi: 10.1093/rheumatology/keae209 (PMC11879323; doi:10.1093/rheumatology/keae209)
Supplement: keae209_Supplementary_Data [file keae209_supplementary_data.docx]

**Supplementary Table S1: Charlson Comorbidity Index Calculation**

| **Original Charlson Comorbidity Index** | | **Adaptation** | |
| --- | --- | --- | --- |
| Item | Score | Item | Score |
| Cerebrovascular disease | 1 | Patient-reported Stroke/TIA* | 1 |
| Congestive heart failure | 1 | LVEF≤50%* | 1 |
| COPD/Asthma | 1 | Patient-reported COPD or asthma | 1 |
| Dementia | 1 | Not recorded; excluded | N/A |
| Depression | 1 | Not recorded; excluded | N/A |
| Hypertension | 1 | Patient-reported hypertension* | 1 |
| Diabetes without end organ dysfunction | 1 | Patient-reported diabetes* | 1 |
| Diabetes with end organ damage | 2 | Not recorded; excluded | N/A |
| Liver disease - Mild | 1 | Not recorded; excluded | N/A |
| Liver disease – moderate or severe | 3 | Not recorded; excluded | N/A |
| Myocardial infarction | 1 | Patient-reported angina or myocardial infarction* | 1 |
| Peripheral vascular disease | 1 | Patient-reported peripheral vascular disease or treatments* | 1 |
| Rheumatic disease | 1 | Applicable to all patients with SSc | 1 |
| Peptic ulcer disease | 1 | Excluded; not recorded independently of other gastrointestinal SSc manifestations | N/A |
| Hemiplegia | 2 | Not recorded; excluded | N/A |
| Moderate to severe renal disease | 2 | Creatinine>265umol/L ever, or previous dialysis or renal transplantation* | 2 |
| Any tumour | 2 | Patient-reported malignancy (excluding NMSC) | 2 |
| Metastatic solid tumour | 6 | Not recorded; excluded | N/A |
| Skin ulcers or cellulitis | 2 | Patient-reported non-hand skin ulcers* | 2 |
| Takes warfarin | 1 | Warfarin or other anticoagulation | 1 |
| Leukaemia | 2 | Patient-reported leukaemia | 2 |
| Lymphoma | 2 | Patient-reported lymphoma | 2 |
| HIV/AIDS | 6 | Not recorded; excluded | N/A |
| **Maximum score** | **38** | **Maximum Score** | **19** |

Abbreviations: AIDS (acquired immunodeficiency syndrome), COPD (chronic obstructive pulmonary disease), HIV (human immunodeficiency virus), LVEF (left ventricular ejection fraction), NMSC (non-melanoma skin cancer), SSc (systemic sclerosis), TIA (transient ischaemic attack), umol/L (micromoles per litre)

**Supplementary Table S2: Sensitivity and specificity calculations**

| **High Risk MUST Score vs. Medium or low risk MUST score** | | | |
| --- | --- | --- | --- |
|  | **High Risk MUST score** | **Low or Medium Risk MUST Score** | Total |
| **GLIM Malnutrition Present** | 471 | 253 | 724 |
| **Not malnourished** | 1 | 812 | 813 |
| Total | 472 | 1065 | 1537 |
| **High Risk MUST Score vs. Low risk MUST score (excluding medium risk scores)** | | | |
|  | **High Risk MUST score** | **Low Risk MUST Score** | Total |
| **GLIM Malnutrition Present** | 471 | 112 | 583 |
| **Not malnourished** | 1 | 555 | 556 |
| Total | 472 | 667 | 1139 |
| **High or Medium Risk MUST Score vs. Low risk MUST score** | | | |
|  | **High or Medium Risk MUST score** | **Low Risk MUST Score** | Total |
| **GLIM Malnutrition Present** | 612 | 112 | 724 |
| **Not malnourished** | 258 | 555 | 813 |
| Total | 870 | 667 | 1537 |
| **Medium vs Low Risk MUST Score (excluding those with high-risk scores)** | | | |
|  | **Medium Risk MUST score** | **Low Risk MUST Score** | Total |
| **GLIM Malnutrition Present** | 141 | 112 | 253 |
| **Not malnourished** | 257 | 555 | 812 |
| Total | 398 | 667 | 1065 |

**High Risk MUST vs. medium or low-risk MUST:**

$$\boldsymbol{Sensitivity=}\frac{\boldsymbol{Disease+\& test+}}{\boldsymbol{true disease}}\boldsymbol{=}\frac{\boldsymbol{471}}{\boldsymbol{724}}\boldsymbol{=65.1\%}$$

$$\boldsymbol{Specificity=}\frac{\boldsymbol{Disease-\& test-}}{\boldsymbol{no disease}}\boldsymbol{=}\frac{\boldsymbol{812}}{\boldsymbol{813}}\boldsymbol{=99.9\%}$$

$$\boldsymbol{PPV=}\frac{\boldsymbol{Disease+\& test+}}{\boldsymbol{all test+}}\boldsymbol{=}\frac{\boldsymbol{471}}{\boldsymbol{472}}\boldsymbol{=99.8\%}$$

$$\boldsymbol{NPV=}\frac{\boldsymbol{Disease-\& test-}}{\boldsymbol{all test-}}\boldsymbol{=}\frac{\boldsymbol{812}}{\boldsymbol{1065}}\boldsymbol{=76.2\%}$$

**High Risk MUST vs. Low risk MUST only:**

$$\boldsymbol{Sensitivity=}\frac{\boldsymbol{Disease+\& test+}}{\boldsymbol{true disease}}\boldsymbol{=}\frac{\boldsymbol{471}}{\boldsymbol{583}}\boldsymbol{=80.8\%}$$

$$\boldsymbol{Specificity=}\frac{\boldsymbol{Disease-\& test-}}{\boldsymbol{no disease}}\boldsymbol{=}\frac{\boldsymbol{555}}{\boldsymbol{556}}\boldsymbol{=99.8\%}$$

$$\boldsymbol{PPV=}\frac{\boldsymbol{Disease+\& test+}}{\boldsymbol{all test+}}\boldsymbol{=}\frac{\boldsymbol{471}}{\boldsymbol{472}}\boldsymbol{=99.8\%}$$

$$\boldsymbol{NPV=}\frac{\boldsymbol{Diseas-\& test-}}{\boldsymbol{all test-}}\boldsymbol{=}\frac{\boldsymbol{555}}{\boldsymbol{667}}\boldsymbol{=83.2\%}$$

**Medium Risk MUST vs. low-risk MUST:**

$$\boldsymbol{Sensitivity=}\frac{\boldsymbol{Disease+\& test+}}{\boldsymbol{true disease}}\boldsymbol{=}\frac{\boldsymbol{141}}{\boldsymbol{253}}\boldsymbol{=55.7\%}$$

$$\boldsymbol{Specificity=}\frac{\boldsymbol{Disease-\& test-}}{\boldsymbol{no disease}}\boldsymbol{=}\frac{\boldsymbol{555}}{\boldsymbol{812}}\boldsymbol{=68.3\%}$$

$$\boldsymbol{PPV=}\frac{\boldsymbol{Disease+\& test+}}{\boldsymbol{all test+}}\boldsymbol{=}\frac{\boldsymbol{141}}{\boldsymbol{398}}\boldsymbol{=35.4\%}$$

$$\boldsymbol{NPV=}\frac{\boldsymbol{Disease-\& test-}}{\boldsymbol{all test-}}\boldsymbol{=}\frac{\boldsymbol{555}}{\boldsymbol{667}}\boldsymbol{=83.2\%}$$

**Supplementary Table S3: Univariable Cox Proportional Hazard Modelling analyses**

| **Variable** | **Hazard Ratio** | **95% Confidence Interval** | **p-value** |
| --- | --- | --- | --- |
| GLIM Malnutrition | 2.0 | 1.6-2.5 | <0.01 |
| Age at SSc onset | 1.1 | 1.1-1.1 | <0.01 |
| Male sex | 2.5 | 1.9-3.1 | <0.01 |
| Diffuse SSc | 1.9 | 1.5-2.4 | <0.01 |

Abbreviations: GLIM (Global Leadership Initiative on Malnutrition), SSc (systemic sclerosis).

**Supplementary Table S4: Sensitivity analysis for survival impact of GLIM Malnutrition using multivariable Cox proportional hazard modelling in participants with incident SSc^&^ only.**

| **Variable** | **Hazard Ratio** | **95% Confidence Interval** | **p-value** |
| --- | --- | --- | --- |
| **Model using GLIM Malnutrition diagnosis (n=704)** | | | |
| GLIM Malnutrition* | 1.6 | 1.1-2.4 | 0.02 |
| Age at SSc onset (years) | 1.1 | 1.1-1.1 | <0.01 |
| Male sex | 2.4 | 1.6-3.6 | <0.01 |
| Diffuse SSc | 2.0 | 1.4-3.0 | <0.01 |
| **Model using BMI (n=704)** | | | |
| BMI <20kg/m^2^* | 1.7 | 1.1-2.6 | 0.02 |
| Age at SSc onset (years) | 1.1 | 1.1-1.1 | <0.01 |
| Male sex | 2.5 | 1.7-3.8 | <0.01 |
| Diffuse SSc | 2.3 | 1.3-2.9 | <0.01 |
| **Model using weight loss (highest recorded, %) (n=581)** | | | |
| Weight loss <5%* | . | . | . |
| Weight loss 5-10%* | 0.8 | 0.4-1.5 | 0.54 |
| Weight loss >10%* | 1.7 | 1.0-2.7 | 0.04 |
| Age at SSc onset (years) | 1.1 | 1.0-1.1 | <0.01 |
| Male sex | 2.4 | 1.5-3.9 | <0.01 |
| Diffuse SSc | 1.9 | 1.2-2.9 | <0.01 |
| **Model using muscle atrophy (n=697)** | | | |
| Muscle atrophy* | 1.5 | 1.0-2.2 | 0.06 |
| Age at SSc onset (years) | 1.1 | 1.1-1.1 | <0.01 |
| Male sex | 2.4 | 1.6-3.7 | <0.01 |
| Diffuse SSc | 1.8 | 1.2-2.7 | <0.01 |

Abbreviations: BMI (body mass index), GLIM (Global Leadership Initiative on Malnutrition), kg/m^2^ (kilograms per meters squared), n (number), SSc (systemic sclerosis). *Denotes ever from SSc onset. ^&^Incident SSc defined as ASCS recruitment within 5 years of SSc onset.

**Supplementary Table S5: Univariable logistic regression analysis of determinants of GLIM Malnutrition**

| **Variable** | **Odds Ratio** | **95% Confidence Interval** | **p-value** |
| --- | --- | --- | --- |
| Age at SSc onset | 1.0 | 1.0-1.0 | <0.01 |
| Male sex | 1.1 | 0.9-1.4 | 0.42 |
| Diffuse cutaneous SSc | 1.4 | 1.2-1.7 | <0.01 |
| ENA Scl70 positivity | 1.1 | 0.8-1.4 | 0.64 |
| Multimorbidity^1^ | 2.0 | 1.6-2.4 | <0.01 |
| Vomiting* | 2.1 | 1.7-2.6 | <0.01 |
| Dysphagia* | 1.7 | 1.4-2.1 | <0.01 |
| Vomiting or dysphagia*^2^ | 2.3 | 1.9-2.8 | <0.01 |
| Diarrhoea* | 1.6 | 1.3-1.9 | <0.01 |
| Faecal incontinence* | 1.7 | 1.4-2.0 | <0.01 |
| Diarrhoea or incontinence*^3^ | 1.6 | 1.4-2.0 | <0.01 |
| Oral Aperture (lowest recorded*, cm)^4^ | 1.3 | 1.2-1.5 | <0.01 |
| Digital ulcers* | 2.0 | 1.7-2.4 | <0.01 |
| CRP>5IU/L* | 1.9 | 1.6-2.3 | <0.01 |
| PAH* | 2.7 | 1.9-3.6 | <0.01 |
| ILD on HRCT* |  |  |  |
| Limited ILD^5^ | 1.4 | 1.1-1.7 | 0.01 |
| Extensive ILD^5^ | 2.3 | 1.7-3.2 | <0.01 |

*Denotes ever from SSc onset. ^1^Multimorbidity defined as Charlson Comorbidity Index Scores ≥4. ^2^Symptoms of patient-reported vomiting or dysphagia combined due to strong association with malnutrition, and strong correlation between variables. ^3^Symptoms of patient-reported diarrhoea or faecal incontinence combined due to strong association with malnutrition, and strong correlation between variables. ^4^Lowest-recorded value of oral aperture centred around mean value to facilitate meaningful odds ratio calculation (mean oral aperture minus measured oral aperture value), to describe the increase in malnutrition risk with each cm below mean oral aperture. ^5^Limited-stage ILD defined as <20% HRCT involvement, or 20-30% involvement with percent-predicted forced vital capacity≥70%, while extensive-stage ILD defined as ≥30% HRCT extent, or 20-30% HRCT involvement with percent-predicted forced vital capacity<70%. **Abbreviations**: confidence interval (CI), cm (centimetre), CRP (C-reactive protein), GLIM (Global Leadership Initiative on Malnutrition), ILD (interstitial lung disease), IU/L (international units per litre), OR (odds ratio), PAH (pulmonary arterial hypertension), SSc (systemic sclerosis).
